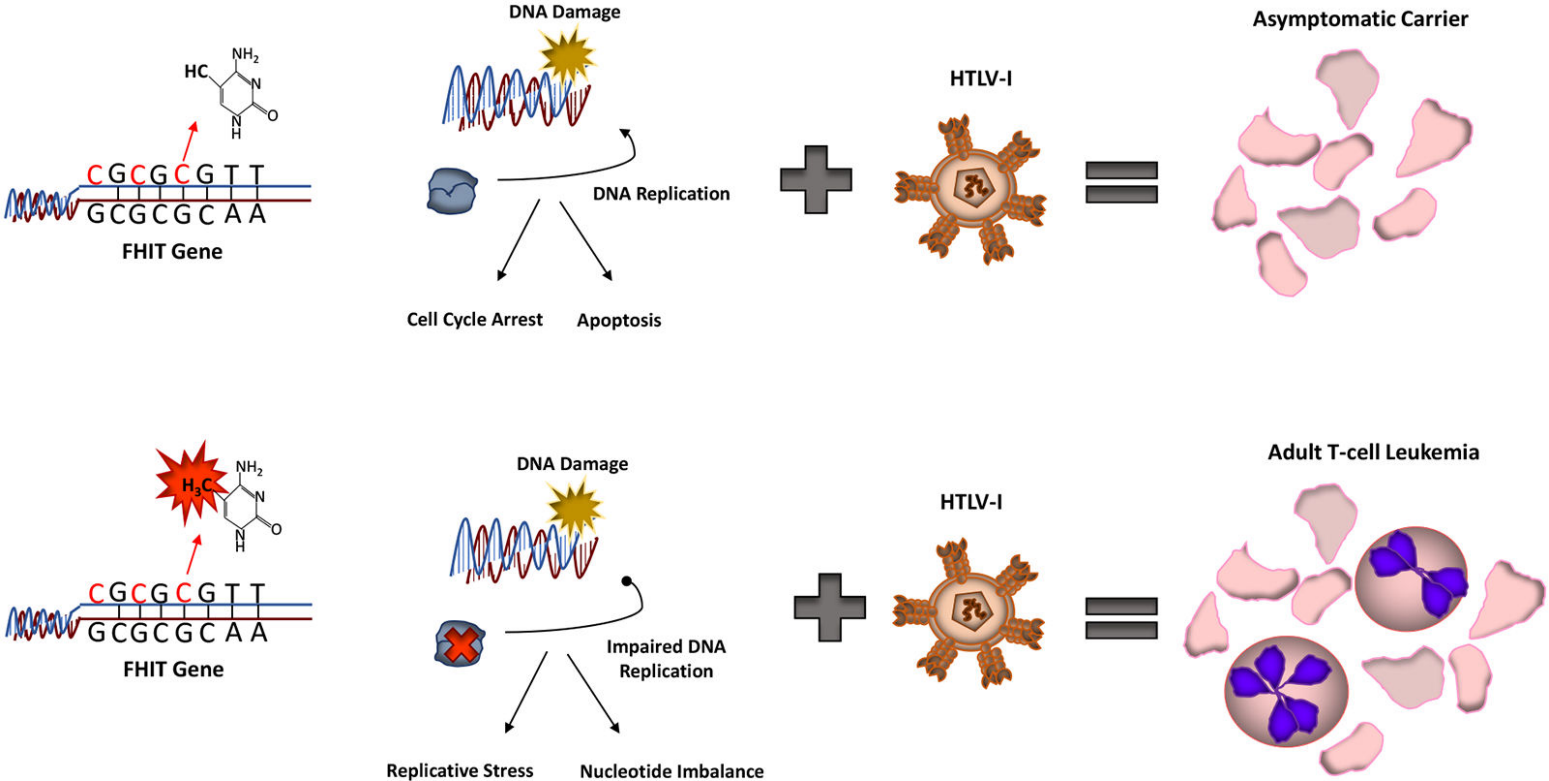# FHIT as a biomarker for early screening of adult T-cell leukemia

**DOI:** 10.46439/cancerbiology.2.028

**Published:** 2021

**Authors:** Marcia Bellon, Christophe Nicot

**Affiliations:** Department of Pathology and Laboratory Medicine. University of Kansas Medical Center, Kansas City, KS, USA

## Abstract

Adult T-cell leukemia (ATL) is an incurable leukemia deriving from human T-cell leukemia virus (HTLV-I) infected cells. In our most recent study, we discovered that methylation of the tumor suppressor, fragile histidine triad gene (FHIT), exists in the majority of acute and chronic ATL patients. Methylation was seen in non-tumorigenic cells, in cells with low levels of HTLV-I integrated DNA, in longitudinal samples from HTLV-I carriers, in a percentage of HTLV-I carriers, and in direct descendants of ATL patients. Overall, this suggests that FHIT methylation is likely present in patients, prior to HTLV-I infection, and predisposes HTLV-I carriers to ATL development. In this commentary we discuss the importance of developing diagnostic tools for the early detection of FHIT methylation and the possibility that prior FHIT methylation may predispose any individual to the development of cancer.

## Introduction

Adult T-cell leukemia/lymphoma is a malignant CD4 T-cell lymphoproliferative disease associated with infection by the retrovirus, human T-cell leukemia/lymphoma virus type-1 (HTLV-I). ATL has a wide-ranging clinical spectrum divided into four clinically distinct diseases: acute, chronic, smoldering and lymphoma. Acute type accounts for nearly 60% of all ATL [1]. For acute and chronic type ATL, the median survival times are 8.3 and 10.6 months, with a 4-year overall survival rates of 11 and 16%, respectively [2]. A major challenge in the treatment of ATL diseases is related to the fact that infection by HTLV-1 is not associated with any particular symptom(s) and as a result most individuals may not know they are infected and at risk of developing leukemia until the disease has advanced to late stages and is least treatable. Care for asymptomatic carriers and smoldering ATL follows the “watch and see” approach to monitor for any signs of disease progression and initiation of treatment. If left untreated, indolent forms of ATL nearly always progresses to more aggressive subtypes, with half of chronic type ATL progressing to acute and dying within 18 months [3]. In contrast, studies have demonstrated a significant extended lifespan for patients with chronic or smoldering that received AZT/IFN treatment. Given the low survival rate and the need to initiate therapy sooner rather than later, the need for accurate and easily detectable diagnostic and prognostic markers is essential.

## Commentary

### FHIT methylation as a diagnostic tool for the development of ATL

Approximately 15-20 million individuals are infected with HTLV-I worldwide and the lifetime risk of developing ATL is 1-5% [4,5]. For decades researchers have attempted to determine what allows some HTLV-I carriers to remain asymptomatic, while others go on to develop ATL. For the past 40 years there has been limited options for a diagnostic tool that could determine the likelihood that an HTLV-I carrier would develop ATL. Most of these markers were either not consistent across a large swath of carriers, were only distinct for certain geographical regions, and/or required extensive analysis that is not conducive as a simple, diagnostic, medical tool. Several of the features that have been linked to the progression of ATL include, high proviral loads, familial history of ATL, age of the patient, oligo- or mono-clonal expansion of infected cells, high plasma levels of soluble tumor necrosis factor receptor 2 (sTNFR2), and mutations in EP300 and the NF-κB/NFAT and TCR/NF-κB pathways [6-10]. In addition, a group of driver mutations that includes PLCG1, PRKCB, CCR4, TP53, and NOTCH1, have been linked with progression to ATL [11]. Despite these advances in understanding ATL progression, none of the markers described above are representative of the vast majority of acute ATL patients and thus no diagnostic test yet exists for the screening of HTLV-I carriers. While studying the role of epigenetic factors in ATL, we discovered that the fragile histidine triad gene, FHIT, was highly and statistically methylated in acute (78%) and chronic (89%) ATL disease [12]. Moreover, HTLV-I infected carriers, and those that presented with TSP/HAM, a neurological disease, rarely carried methylated FHIT. Strikingly, FHIT methylation was not only found in tumorigenic T-cells, but also in cells and tissue of non-tumor origin. FHIT methylation also did not correlate with the viral load of the patient, meaning that even in acute and chronic ATL patients with low HTLV-I proviral loads, FHIT methylation was still present. This data alone demonstrates that FHIT methylation represents a clear distinction between acute/chronic ATL disease and non-ATL/HTLV-I infected people. Strikingly, we also discovered that nearly 6% of HTLV-I carriers had a methylated FHIT gene. This closely correlates with the known fact that around 3-5% of asymptomatic HTLV-I carriers go on to develop ATL. Moreover, in the study we reported that direct descendants of ATL patients were found to possess a methylated FHIT gene, regardless of disease status. Longitudinal studies following FHIT methylation are the next step in validating these results. However, early indicators suggest that in those HTLV-I carriers that possess FHIT methylation, these patients will develop ATL disease in the next 20-40 years. In support of this conclusion, the study demonstrated that in asymptomatic carriers, FHIT methylation was already present in patients that would develop ATL in less than 2 years’ time. In fact, these patients looked very much like smoldering type ATL patients, even though they were still asymptomatic and had no clinical evidence of leukemia. This strongly supports the idea that FHIT gene methylation can be used as a predictive biomarker for ATL disease progression. The data clearly advocates that early testing of FHIT methylation status can lead to the determination of ATL leukemia risk assessment perhaps years or decades before the disease is initiated.

### Does pre-existing FHIT methylation contribute to tumorigenesis?

An interesting finding from our study was that a small percentage (over 5%) of PBMCs from normal, otherwise healthy donors, presented with methylated FHIT. As described in our study, pre-existing FHIT methylation predisposes HTLV-I carriers to the development of ATL (Figure 1). However, a more global question, is whether FHIT methylation inclines any individual to a wide array of cancers, not just ATL? It has already been suggested that some environmental factors, such as smoking can influence FHIT methylation [13]. Whether FHIT methylation is the ultimate factor predisposing these individuals to carcinomas is unknown. Along with our data, there is existing evidence that FHIT methylation occurs in normal adult cells and tissue. In support of this, and outside our own study, FHIT has been found methylated in normal whole blood samples. In these samples, up to 25% of healthy age-matched whole blood samples from children (5.6 +/−2.9 years) with ALL carried methylated FHIT [14]. Given that FHIT is found methylated in a small percentage of cells derived from PBMCs, there is a possibility that prior FHIT methylation may predispose individuals to leukemia/lymphomas. For other cancers, FHIT may be inactivated through other means. In fact, altered sequence patterns and FHIT instability have already been seen in normal cells, in the absence of replicative stress [15]. It is already known that genetic disruption of the FHIT gene, by means of allelic deletion, is found in large portion of lung cancer patients [16]. It is also likely, given the importance of FHIT loss in cancer, that transcriptional factors, lncRNAs, mRNAs, or circRNAs affect FHIT expression and remain to be identified.

The importance of FHIT methylation in solid cancers has been documented. FHIT was found methylated not only in primary lung tissue samples from the center of non-small cell lung cancer (NSCLC) lesions but also the operational margin, a normal area surrounding the primary lesion [17]. Meta-analysis studies of methylation in NSCLC patients and normal patients have shown similar, albeit highly inconsistent findings on FHIT methylation in normal samples [18,19]. Though FHIT methylation was significantly higher in primary NSCLC samples, the level of FHIT methylation in control samples varied widely. Among studies, anywhere from 5.4-13.4% of normal lung tissue was methylated for FHIT, compared to 30.8-32.1% for NSCLC [18]. An important consideration is whether the methylation of the normal tissue is from contamination from the underlying tumor. To alleviate this concern, in our study, we analyzed cells deriving from non-tumorigenic T-cells, B-cells, and nail samples from the same patient. All samples were found to harbor FHIT methylation. However, often primary lung and breast cancer samples are paired – the test sample originating from the center of the solid tumor itself and the control taken from an area outside the tumor margin, which is macroscopically deemed “normal”. The inconsistent readings between studies and the high levels of FHIT methylation in normal tissues deriving from these solid tumors could be due to contamination with surrounding, tumorigenic tissue. A study found FHIT methylation in non-neoplastic adjacent tissue in 8.3% of lung and 14% of breast tissue [20]. However, when normal breast tissue was analyzed, no FHIT methylation was seen.

A consideration for future studies is that the level of FHIT methylation in the public is likely to be low. In our study, only 5% of PBMCs had methylated FHIT. Therefore, studies would need to sample a significantly large cohort of normal tissue or cells to see if FHIT methylation is truly found in correspondingly healthy cells/tissue. Another possibility is that perhaps the “normal” tissue, outside the surgical margins, is truly FHIT methylation positive, and not due to contamination from the surrounding lesion. If so, even though this tissue presents as normal, FHIT methylation may already be present, suggesting that FHIT methylation may be one of the earliest markers for tumor development. FHIT methylation may serve as the initial event that triggers cellular transformation. This is not improbable given the role of FHIT as a tumor suppressor and genome caretaker. Loss of FHIT has already been shown to induce genome instability and provides single-strand DNA substrates for hypermutation [21]. If this is the case, FHIT methylation could help determine if surrounding tissue (which macroscopically appears normal) may be susceptible to transformation and the spreading of the tumor. If physicians could analyze the surrounding tissue for FHIT methylation, it will allow them to know if greater margins need to be excised to prevent future tumor outgrowth. Currently, no clinical test for FHIT methylation exists. However, methylation as a diagnostic tool has already been used in the clinical setting. Cologuard^©^, Epi proColon^©^, and EarlyTect^©^ are non-invasive screening tests for colon cancer that are currently in use and rely on the methylation of genes such as BMP3, NDRG4, SEPT9, SDC2 for early detection [14]. Furthermore, methylation-based screening tests for hepatocellular carcinoma, lung, bladder, prostate, and cervical cancers are in development, demonstrating the practicality of methylation-based diagnostic tools for early screening or disease progression. These data clearly call for the development of ATL screening tests based off FHIT methylation.

## Conclusion

FHIT methylation serves as a robust biomarker signature for the progression of acute and chronic ATL disease. The development of simple, diagnostic tests to screen asymptomatic, HTLV-I carriers could provide early treatment options and attenuate the development of aggressive forms of ATL. Given the role of FHIT in early cancer initiation, it will be exciting to see if diagnostic tools for FHIT methylation could play a larger role in determining initial cancer development.

## Figures and Tables

**Figure:**